# The criticality Index-mortality: A dynamic machine learning prediction algorithm for mortality prediction in children cared for in an ICU

**DOI:** 10.3389/fped.2022.1023539

**Published:** 2022-12-01

**Authors:** Anita K Patel, Eduardo Trujillo-Rivera, Hiroki Morizono, Murray M. Pollack

**Affiliations:** ^1^ Department of Pediatrics, Division of Critical Care Medicine, Children's National Health System, George Washington University School of Medicine and Health Sciences, Washington, DC, United States; ^2^ Department of Bio-Informatics, Children's National Health System, George Washington University School of Medicine and Health Sciences, Washington, DC, United States; ^3^ Department of Pediatrics, Children's National Research Institute, George Washington University School of Medicine and Health Sciences, Washington, DC, United States

**Keywords:** prediction algorithm, dynamic outcome prediction, mortality prediction, criticality index, machine learning

## Abstract

**Background:**

The Criticality Index-Mortality uses physiology, therapy, and intensity of care to compute mortality risk for pediatric ICU patients. If the frequency of mortality risk computations were increased to every 3 h with model performance that could improve the assessment of severity of illness, it could be utilized to monitor patients for significant mortality risk change.

**Objectives:**

To assess the performance of a dynamic method of updating mortality risk every 3 h using the Criticality Index-Mortality methodology and identify variables that are significant contributors to mortality risk predictions.

**Population:**

There were 8,399 pediatric ICU admissions with 312 (3.7%) deaths from January 1, 2018 to February 29, 2020. We randomly selected 75% of patients for training, 13% for validation, and 12% for testing.

**Model:**

A neural network was trained to predict hospital survival or death during or following an ICU admission. Variables included age, gender, laboratory tests, vital signs, medications categories, and mechanical ventilation variables. The neural network was calibrated to mortality risk using nonparametric logistic regression.

**Results:**

Discrimination assessed across all time periods found an AUROC of 0.851 (0.841–0.862) and an AUPRC was 0.443 (0.417–0.467). When assessed for performance every 3 h, the AUROCs had a minimum value of 0.778 (0.689–0.867) and a maximum value of 0.885 (0.841,0.862); the AUPRCs had a minimum value 0.148 (0.058–0.328) and a maximum value of 0.499 (0.229–0.769). The calibration plot had an intercept of 0.011, a slope of 0.956, and the *R^2^* was 0.814. Comparison of observed vs. expected proportion of deaths revealed that 95.8% of the 543 risk intervals were not statistically significantly different. Construct validity assessed by death and survivor risk trajectories analyzed by mortality risk quartiles and 7 high and low risk diseases confirmed *a priori* clinical expectations about the trajectories of death and survivors.

**Conclusions:**

The Criticality Index-Mortality computing mortality risk every 3 h for pediatric ICU patients has model performance that could enhance the clinical assessment of severity of illness. The overall Criticality Index-Mortality framework was effectively applied to develop an institutionally specific, and clinically relevant model for dynamic risk assessment of pediatric ICU patients.

## Introduction

Models computing mortality risk have not been traditionally used for individual patient risk assessment. Static scores such as PRISM and PIM that utilize fixed time periods for data collection at the beginning on the intensive care unit stay (ICU) stay were developed and calibrated on populations of interest primarily for hospital quality assessment and benchmarking; they were not designed to aid in the assessment of individual patients or to enhance clinical decision making (1, 2). Since they are calibrated to produce a risk score at a single time point, they fail to provide updated assessments reflecting the changing clinical status of individual patients; some have warned against their use for individual patients (3–5). When the data are collected or risk is computed at the bedside, there are data reliability issues as well as an additional risk of human error. However, there is a long-standing desire to have a frequently updated patient assessment methodology as evidenced by investigations of intermittently updated static scoring systems (6–10).

Physiologic patterns often precede acute deterioration of patients and may go unrecognized (11–15). This is the foundation of the recent emphasis on early warning systems (15). An automated, frequently updated assessment of a patient's severity of illness could alert health care providers to a patient's changing status prior to clinical recognition (16). For ICU patients, serious deteriorations often follow a sequence of both deteriorations and improvements until there is overt decompensation, rather than a linear progression to decompensation (14). Alerts that identify an unrecognized problem or reinforce a provider's suspicions could result in monitoring or therapy changes that mitigate mortality and morbidity risks.

Despite the increasing sophistication of monitoring and therapeutic technologies, and the data-rich environment of the ICU, current monitoring strategies in clinical use are still challenged to accurately assess changing patient status. The aim of this study was to assess the performance of a dynamic method of updating mortality risk every 3 h using the Criticality Index-Mortality (CI-M) neural network methodology.

## Methods

The data came from Children's National Hospital, a 323-bed academic hospital with 48 bed Pediatric ICU beds and 24 Cardiac ICU beds. The patient sample included all admissions to the Pediatric and Cardiac ICUs from January 1, 2018 to February 29, 2020. The end date was chosen to reflect care prior to the novel coronavirus pandemic since ICU admission and discharge guidelines and isolation practices were in flux for much of this time. Exclusions included patients over 21 years of age and those admitted to the neonatal ICU. This study was approved by the Institutional Review Board (protocol Pro00015931). Modeling was performed under the direction of Eduardo Trujillo-Rivera PhD.

### Dataset

The dataset was extracted from the electronic health record (EHR). Descriptive data included age, gender, dates of hospital and ICU admission, admission type (with elective, emergent, and urgent), diagnoses, race, and hospital outcome (survival, death) during or following an ICU admission. Only data from the first ICU admission was included for patients with multiple admissions. Modeling included all independent variables from the original multi-institutional CI-M model representing physiology, treatment, and care intensity.(Trujillo Rivera et al., 2022)17 This included age, gender, 30 laboratory tests, 6 vital signs, 1113 and medications classified into 143 categories. Positive pressure ventilation (invasive and non-invasive) was identified by procedure codes, and specific times with and without ventilation were identified. Variables reflecting positive pressure ventilation included the number of hours of mechanical ventilation at the time of risk assessment, the number of hours since discontinuation at the time of risk assessment, and the proportion of time that positive pressure ventilation used. Medication data were extracted from the medication administration record using start and end times. Medication classes were categorized using the Multum™ system (18). A comprehensive list of independent variables and details of medication classification have been published and included in Supplementary Appendices S1, S2 (17). Other data included diagnostic information categorized by the International Classification of Diseases 10th Edition (ICD-10) (19). Diagnoses were used for descriptive purposes, but not for modelling, because they were determined at discharge. All discharge diagnoses for each patient were categorized by system of dysfunction (Table 1).

**Table 1:** **Population characteristics** the mann whitney U test was used to compare the distribution of numerical quantities between survivors and deaths. The Santner and Snell exact test was used to compare proportions (percentages) between survivors and deaths. A single encounter could have diagnoses in multiple groups.

Characteristic	All	Survivors	ICU Deaths	*P*-values^d^
N	8,399	8,087	312	
Age (Months)^a^	38 (12, 130)	37 (11,130)	50 (1,132)	0.034
Female (*n* (%)	3,789 (45.1)	3,650 (45.13)	139 (44.55)	0.841
Hospital LOS (hours)^a^	83.10 (52.33,151.79)	82.50 (51.88,147.06)	143.97 (65.78,323.60)	<0.001
ICU LOS (hours)^a^	35 (20, 69)	34 (20, 67)	78 (43, 178)	<0.001
Admission Type^b^				
Elective	2,450 (29.2)	2,399 (29.66%)	51 (16.35%)	<0.001
Emergency	5,666 (67.5)	5,427 (67.11%)	239 (76.60%)	<0.001
Urgent	282 (3.4)	261 (3.23%)	21 (6.73%)	0.225
Hospital Mortality	312 (3.7)			
Positive Pressure Ventilation, *n* (%)	3,118 (37.1)	2,882 (35.64)	236 (75.64)	<0.001
Congenital Disease *n* (%)	1,975 (23.5%)	1,891 (23.4%)	84 (26.8%)	0.243
Systems of Dysfunction^c^ *n* (%)				
Respiratory	4,781 (56.9%)	4,563 (56.4%)	218 (69.4%)	<0.001
Endocrine, Nutritional, Metabolic, and Immune	1,343 (16.0%)	1,249 (15.4%)	94 (29.9%)	<0.001
Gastrointestinal	843 (10.0%)	770 (9.5%)	73 (23.2%)	<0.001
Infectious	1,127 (13.4%)	1,052 (13.0%)	75 (23.9%)	<0.001
Injury and Poisoning	971 (11.6%)	898 (11.1%)	73 (23.2%)	<0.001
Neurological	2,177 (25.9%)	2,034 (25.2%)	143 (45.5%)	<0.001
Neoplasms	434 (5.2%)	377 (4.7%)	57 (18.1%)	<0.001
Hematologic	609 (7.3%)	541 (6.7%)	68 (21.7%)	<0.001
Cardiovascular	1,235 (14.7%)	1,113 (13.8%)	122 (38.9%)	<0.001
Musculoskeletal	663 (7.9%)	636 (7.9%)	27 (8.6%)	0.799
Dermatologic	359 (4.3%)	330 (4.1%)	29 (9.2%)	0.073
Genitourinary	516 (6.1%)	455 (5.6%)	61 (19.4%)	<0.001
Ophthalmologic	238 (2.8%)	210 (2.6%)	28 (8.9%)	0.0286
ENT	182 (2.2%)	181 (2.2%)	1 (0.3%)	0.504
Psychiatric	568 (6.8%)	540 (6.7%)	28 (8.9%)	0.437

LOS, length of stay.

^a^ Median (25^th^ percentile, 75^th^ percentile).

^b^ Emergency refers to all admissions/transfers that are classified as needing the ICU. Urgent refers to all patients who were intubated or actively decompensating that required immediate transfer/admission to the ICU. These metrics were supplied for descriptive purposes, and not used for modeling.

^c^ A single encounter could have diagnoses in multiple groups.

^d^ Survivors and deaths were compared using univariate analysis.

The ICU course was discretized into consecutive 3-hour time periods truncated at 11.4 days (273 h) when the sample size was reduced to 97 survivors and 10 deaths to ensure an appropriate sample for calibration testing. Deaths could have occurred during or following the ICU admission; however, they had to occur during the specific hospital admission to be included as a death in the study population presented in Table 1. We randomly selected 75% of patients for training, 13% for validation, and 12% for testing. The training set was used for model development, the validation set was used to fine-tune parameters and prevent over-fitting, and the test set was used to evaluate model performance. Counts of survivors and deaths per 3-hour time period in the training/validation and test set are provided in Supplementary Appendix S3 for reference.

Vital signs, laboratory data, and medication data were standardized to values from 0 to 1 using the maximum and minimum values of the training set. Consistent with other machine learning models, the data for each time period were forward imputed using the last available data if new data were not obtained (16, 17, 20–23) For the first time period, if vital signs or laboratory data were not obtained, we used the medians of the first time periods across all training patients adjusted by 9 age groups. The imputed values by age groups are reported in Supplementary Appendix S4. For modeling, imputed values were identified by setting the count to zero. Vital signs and laboratory values for each time period were summarized using the count, averages, standard deviations, maximums, and minimums. Medication administration data included the number of medications by class but not dose. Medication history was summarized in each time period by the number of medications in a class, the proportion of time periods the medication class was previously administered, and the number of administered medications within the class.

### Machine learning methodology

A single neural network for classification of hospital outcome as survival or death was developed. The model used variables up to 6 h in the past for all patients if these data were available. The objective of each training epoch was to maximize the Mathew's correlation coefficient at a cut point of 0.5 while minimizing the binary cross entropy between the predicted score and the patient's outcome. The initial neural network had a single hidden layer, and a logit output. We consecutively increased the number of nodes while monitoring the Mathew's correlation coefficient, sensitivity, specificity, precision, and negative predictive value at cut points of 0.15, 0.5, and 0.9 in the training and validation sets. If overfitting was detected, regularization included L2 regularization and layer node dropouts with parameters tuned to maintain similar metrics on the training and validation sets. We increased the number of nodes while monitoring for overfitting. When there were no additional gains on the performance metrics, we added another hidden layer and repeated the process, increasing the number of hidden layers until our regularization attempts were unsuccessful in avoiding overfitting. The best model as measured by the Mathew's correlation coefficient without overfitting was kept. We assessed models using up to four previous time periods and found that performance was maximized with the current time period and the change between the present and previous time period.

The final model had three hidden layers with 32 nodes each, bias and ReLU activation functions, L2 regularization with a parameter 0.02, and drop out nodes of 0.05. The neural network was calibrated to risk of mortality using a logistic regression with covariates including the logit transformed score of the neural network.

### Statistics

The performance was assessed in the test sample and only test sample data are reported. Discrimination was assessed by the area under the receiver operating characteristic curve (AUROC) and the area under the precision recall curve (AUPRC) for all time periods combined and as function of time (24). AUPRC was computed with integral approximation. The model was calibrated using the training and validation datasets. Calibration was assessed on the test dataset with calibration plots of the differences between the observed and expected proportions of deaths in the risk intervals. The composite calibration plot had 543 risk intervals with a minimum of 49 patients in each risk interval. We computed the regression line for observed vs. expected proportions of deaths and tested the difference between proportions in each interval with the Santner and Snell exact test and report the percentage of intervals with no statistical difference (25, 26). Optimal calibration plot performance includes intercept = 0, slope = 1, *R*^2 ^= 1, and ≤5.0% of risk intervals with a statistically difference (*p* < 0.05) between the observed and expected proportions. In addition, we computed the additional performance metrics of precision, number needed to evaluate, accuracy, and negative predictive value at a cut point of 0.5 and performed a net benefit analysis (27) for the proposed model of both treated and untreated patients.

Construct validity was assessed by plotting population trajectories and mortality risk changes in consecutive time periods for selected groups. First, we plotted the mortality risk trajectories for survivors and deaths in the total sample for specific mortality risk groups. Mortality risk groups were determined from the first 3-hour time period using mortality risk quartiles. A priori based on analyses of clinical trajectories from the national Criticality Index-Mortality model (28), we expected that the mortality risk of deaths in the highest risk quartile would remain high and other death groups would increase or remain relatively constant over time and the mortality risk of survivors in the highest survivor risk quartile group would decrease and the risk of other survivor risk groups would decrease or remain relatively constant over time. Second, we plotted the mortality risk trajectories using diagnostic groups with traditionally low (diabetic ketoacidosis, bronchiolitis) and high risk (congenital cardiovascular conditions, traumatic brain injury, bone marrow transplant, pediatric acute respiratory distress syndrome). We hypothesized that deaths in the low-risk diagnoses would follow a similar clinical trajectory to deaths in the low-risk quartile – they would increase overtime, while survivors would maintain relatively low and constant mortality risks. Whereas, deaths in the high-risk diagnoses would rapidly increase their mortality risks or begin at a high mortality risk and remain high, while survivors' mortality risk trajectories would remain relatively constant or improve over time (28).

### Variable importance

We explored the variable importance using a Local Interpretable Model-Agnostic Explanation (LIME) approach (LIME R package) (29). Briefly, the LIME approach assumes that every model performs like a linear prediction model for each prediction and the hierarchy of covariate importance is preserved. The collection of individual linear models provides an interpretation of the covariate importance in the final model. We ranked the most important variables across all predictions by computing the percentage of times each variable was among the 30 most important covariates.

## Results

There were 8,399 pediatric ICU admissions with 312 (3.7%) deaths (Table 1). Admissions to the ICU were 29.2% elective, 67.5% emergent, and 3.4% urgent. The most common systems of dysfunction were respiratory, cardiovascular, neurologic, infectious, and endocrine/nutrition/metabolic/immune. Median age was 38.1 months (IQR 11.6–129.9), 45.1% were female; median hospital length of stay (LOS) was 83.1 (IQR 52.3–151.8), ICU LOS was 35.3 (IQR 20.01–69.1), and 37.1% of patients received positive pressure ventilation. Deaths were more likely to be older, emergent admissions with longer LOS's, and receive positive pressure ventilation.

**Figure 1:**
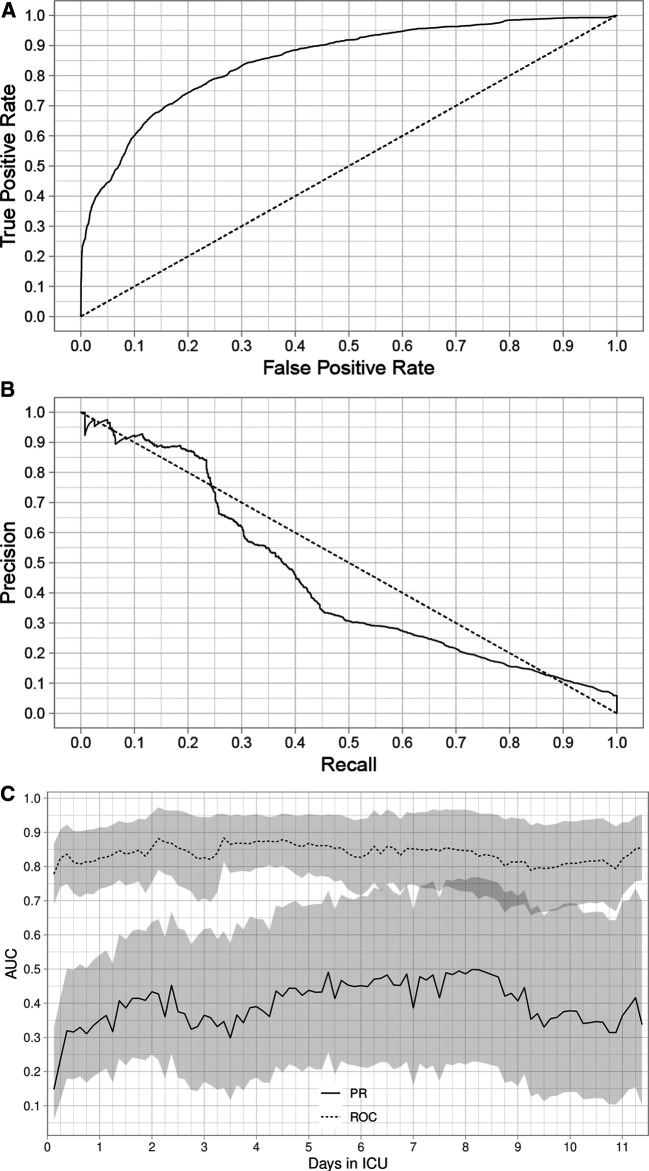
Area under the receiver operating characteristic (AUROC) curves (A), area under the precision recall (AUPRC) curves (B), and AUROC and AUPRC computed every 3 h. Outcomes were survival and death. (**A**) The AUROC for all time periods through 11.4 days. (**B**) The AUPRC for all time periods through 11.4 days. (**C**) AUROC and AUPRC calculated every 3 h through 11.4 days. The shaded regions are pointwise 95% confidence intervals. We applied boost strap techniques to the test set with 5,000 stratified bootstrap replicates to compute the confidence intervals.

**Figure 2:**
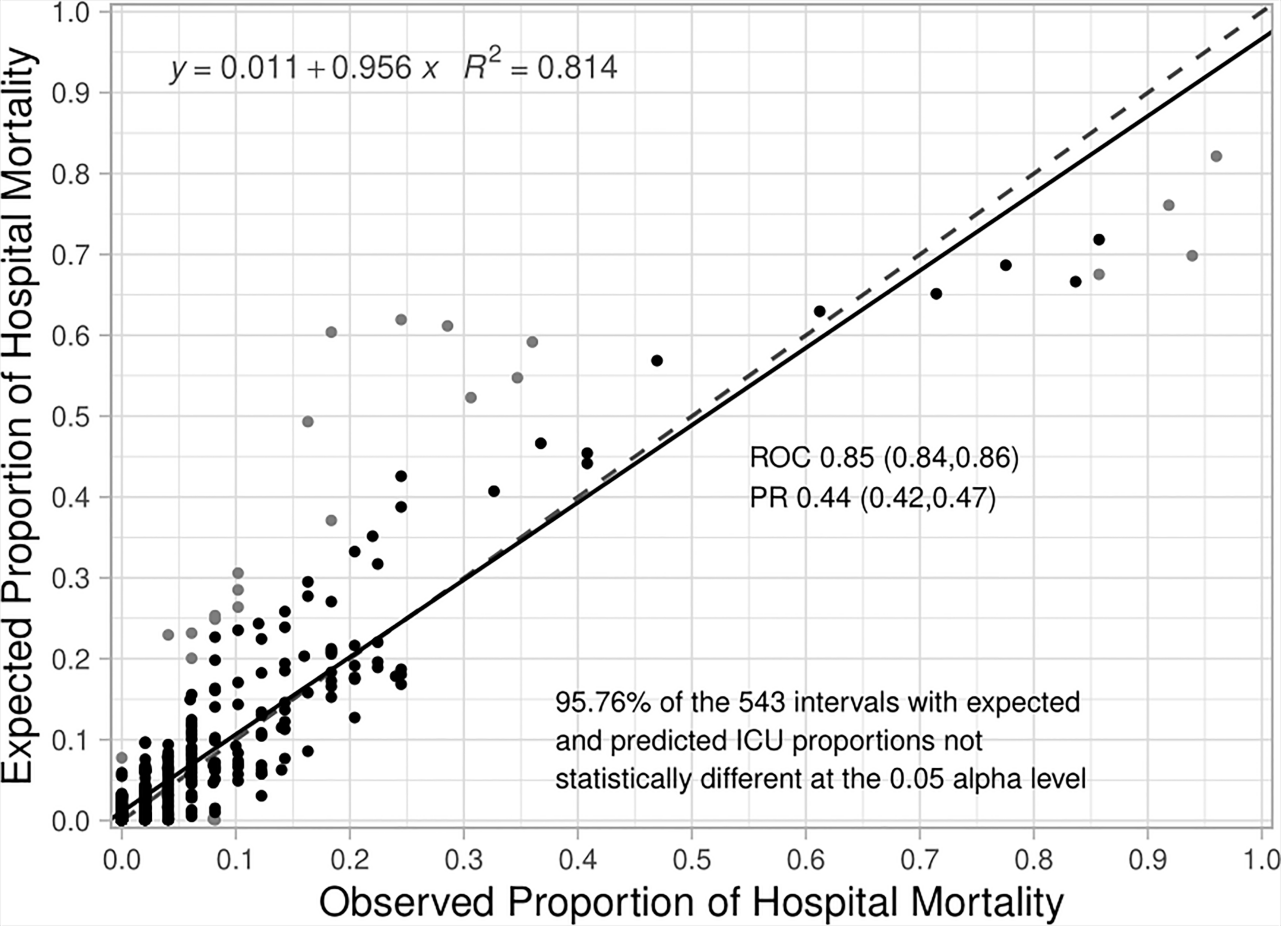
**Calibration plot for the criticality Index-mortality (CI-M) model**. The dashed line is the line of identity and the solid line is the regression line. A statistical test for differences of proportion was computed for each dot in the calibration plot. Each dot is associated with two proportions in a risk interval: 1) observed proportion of ICU mortality (observed count of deaths/total sample), and 2) expected proportion of ICU mortality (predicted count of deaths/total sample). The expected proportion is computed using the risk predictions from the model to predict the count of deaths, the observed proportion is an empirical count of the risk of mortality among the cases in each risk interval. The Santner and Snell test were computed to assess the significance of the different proportions. The dot is grey if the test results in a *p*-value < 0.05.

The composite assessment of discrimination across all time periods found an AUROC of 0.851 (0.841–0.862) and a AUPRC was 0.443 (0.417–0.467) (Figure 1A,B respectively). The AUROCs and AUPRC (Figure 1C) were also calculated for every 3-hour time period. The AUROCs had a minimum value of 0.778 (0.689–0.867) at hour 3 and a maximum value of 0.885 (0.841,0.862) at hour 81 and the individual AUPRCs had a minimum value 0.148 (0.058–0.328) at hour 3 and a maximum value of 0.499 (0.229–0.769) at hour 195. Additional performance metrics are reported in Supplementary Appendix S5, Table J. Most notable is the precision of the model at 0.594, with a number needed to evaluate of 1.7, accuracy of 94.7% and negative predictive value of 0.958. The net benefit analysis also demonstrates the potential positive impact (Supplementary Appendix S6: Net Benefit Analysis of the Criticality Index-Mortality Models for both Treated and Untreated Patients).The calibration plot had an intercept of 0.011, a slope of 0.956, and the *R*^2^ was 0.814 (Figure 2). Comparison of the observed vs. expected proportion of deaths in the risk intervals revealed that 95.8% of the 543 risk intervals were not statistically significant (Figure 2).

**Figure 3:**
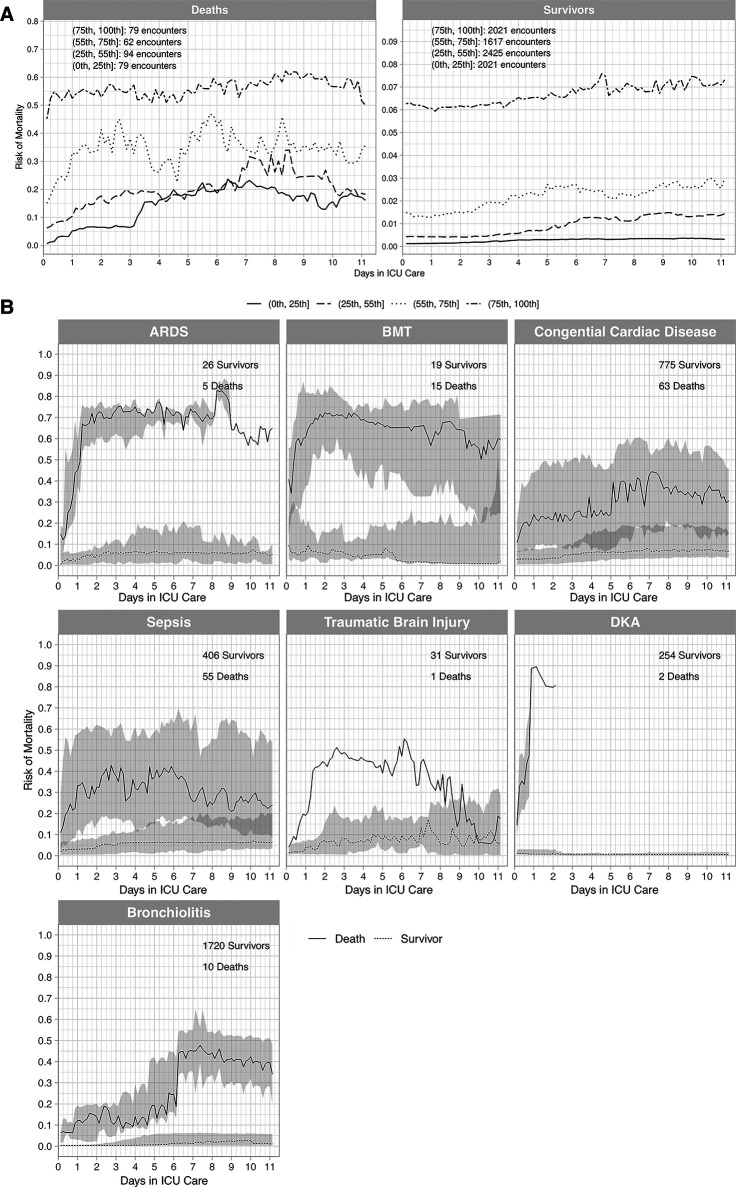
Trajectories for deaths and survivors by risk quartiles (A), and trajectories for deaths and survivors by diagnostic groups (B). (A) Death and survivor risk trajectories. Risk was computed with the Criticality Index-Mortality (CI-M). Median risk for each quartile for survivors and deaths used CI-M predictions. Patients were stratified using the first risk prediction at 3 h. The shaded areas are 95% confidence intervals (CIs). The trajectories were constructed from the total sample with 314 deaths and 8,087 survivors. (B) Death and survivor risk trajectories for different conditions. Risk was computed with the CI-M predictions and displayed for deaths and survivors. The numbers of survivors and deaths are shown in each panel. ARDS = Acute Respiratory Distress Syndrome (ARDS), BMT = Bone Marrow Transplants, DKA = Diabetic Ketoacidosis (DKA).

Construct validity assessed by death and survivor risk trajectories (Figure 3A) revealed the *a priori* expectations of risk trajectories were correct (Methods). Risks for deaths in the highest risk quartile were high and remained high and risks for deaths in the other 3 risk quartiles increased over time All survivor risk quartiles had mortality risks that remained relatively constant over time. Construct validity assessed by the mortality risk trajectories of different disease pathologies (Figure 3B) followed the expected pattern of deaths having higher mortality risks than survivors. Mortality risk trajectories exhibited different patterns across differing pathologies that were consistent with clinical experience.

**Figure 4:**
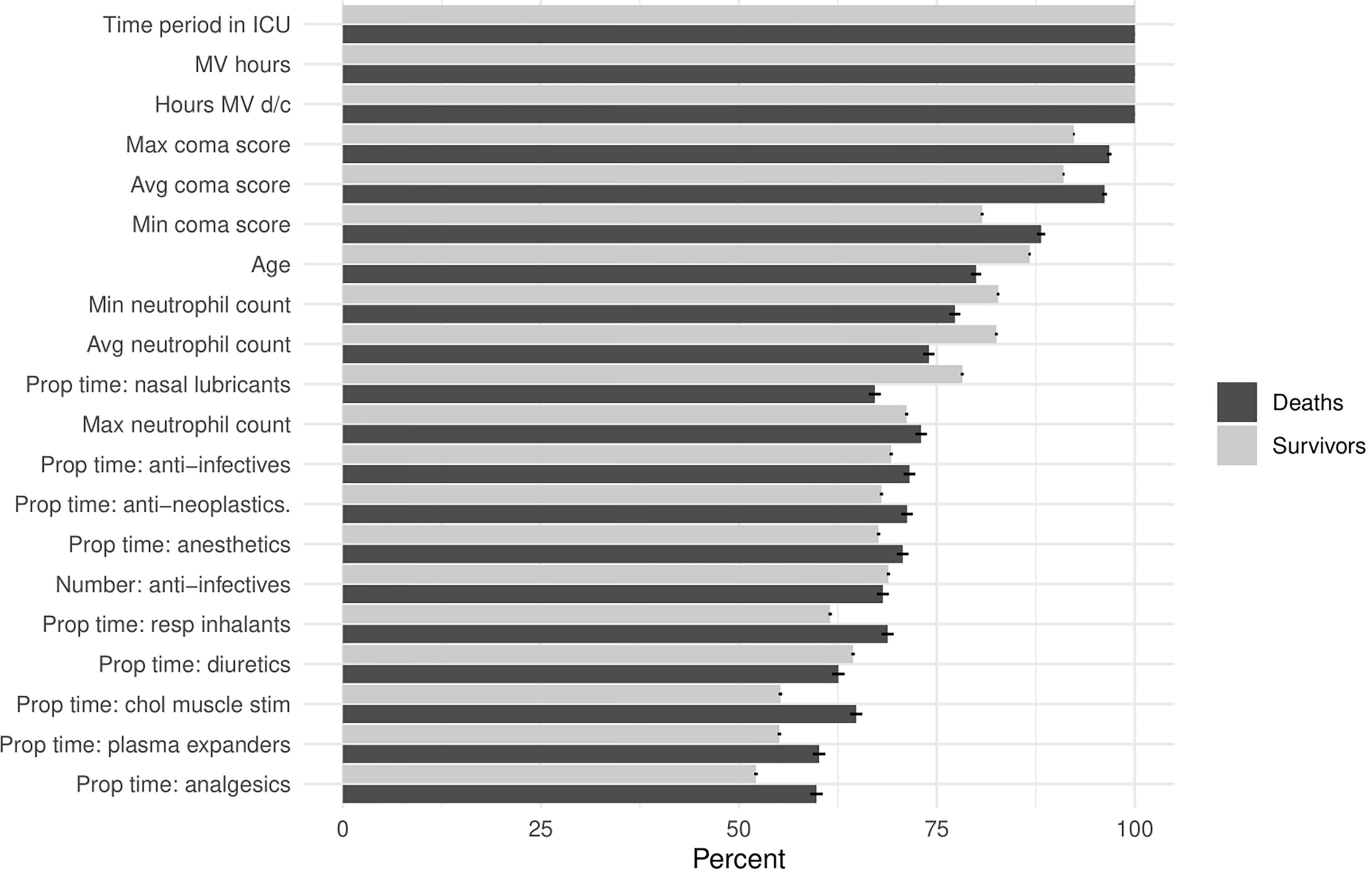
**Variable importance for the Criticality Index-Mortality (CI-M) model**. Percentages of the most frequently important covariates determined by the LIME methodology. MV = mechanical ventilation, d/c = discontinued, max = maximum, avg = average, min = minimum, prop = proportion, resp = respiratory, chol = cholinergic, stim = stimulants.

The 20 most frequently important covariates for risk prediction using the LIME approach are shown in Figure 4. The 10 most frequently important variables were the length of time in the ICU prior to the risk prediction, duration since mechanical ventilation was discontinued, hours on mechanical ventilation, the maximum, minimum and average coma scores, age, and the minimum, average, and maximum neutrophil counts. Other variables of frequent importance included medication classes (anti-infectives, anti-neoplastics, general anesthetics, respiratory inhalants and nasal lubricants). Overall, these variables reflect disease pathologies such as neurologic conditions (coma scores), respiratory conditions (mechanical ventilation, respiratory inhalants, and lubricants).

## Discussion

The CI-M, a neural network which uses physiology, therapy, and intensity of care to compute morality risk for pediatric ICU patients, was effectively used to develop an institution-specific and clinically relevant model for mortality risk assessment every 3 h. Model performance, while it varied over time, indicated potential to enhance clinical assessment of mortality risk in the PICU. Discrimination assessed across all time periods found an AUROC of 0.851 (0.841–0.862) and a AUPRC was 0.443 (0.417–0.467). The calibration plot had an intercept of 0.011, a slope of 0.956, and the *R^2^* was 0.814. Comparison of the observed vs. expected proportion of deaths in 543 the risk intervals revealed that 95.8% were not statistically significant. Construct validity assessed by death and survivor risk trajectories analyzed by mortality risk quartiles and by 7 high and low risk diseases confirmed risk patterns consistent with clinical expectations. Importantly, the worst performance was during the initial time intervals when historical data was less available, and during the later times when less data was available for modeling. These issues would be amenable to improvement as more institutional data, including patients and patient variables, are acquired. Additionally, since the evaluation cohort is constantly changing as patients leave the ICU, a larger data set would also enable a more complete assessment of performance, including the characteristics of patients remaining in the ICU at different time points coupled with focused performance metrics for those time points.

The approach taken to develop an institutional-specific model was straightforward and applicable to institutions with a range of data sciences expertise, including those without research expertise. We used the core variable list and modeling methodology used to develop the CI-M. Since the original CI-M was developed from a national database with a limited number of data elements, we were able to make minor enhancements to the dataset relevant to our institution. Developing an institution specific model also enabled us to fine-tune risk computations to outcomes that might have been influenced by institution-specific clinical practice (30–32). Developing institution-specific models has the further potential advantage for “continuous improvement” by adjusting variables and outcomes as institution-specific practices change and adding variables to improve performance.

While neither current risk assessment nor prediction methods have significantly enhanced the ability of bedside caregivers to recognize early patterns of deterioration (33), the methodology described in this article has the potential to identify patients by their changing risk status who are deteriorating, improving, or remaining stable. If successful, it could improve clinical decision-making by supplementing the limitations of cognitive processing and by reducing medical errors (34). Medical errors are often based in heuristics and are more likely to occur in high-pressure, high-stakes decisions, particularly when dealing with incomplete information, such as assessing a deteriorating patient (35–37). The response to risk change will need to be individualized based on the patient's condition, clinical expectations, and the magnitude of the change. For example, some patients will need immediate evaluation with a change in therapeutic plans while others may be considered for ICU discharge because of the documented improvement.

Health care personnel benefiting most from measures of risk change are expected to be those with less experience or training. Experienced intensivists are excellent at assessing patients using clinical snapshots and their risk assessments for discharge outcomes are generally accurate and reliable (38–40). The wide-spread use of the multiple static models used to estimate objective mortality risks (2, 41, 42) might have helped to calibrate clinical assessments to objective risk predictions for discharge outcomes. Unfortunately, even experienced physicians' clinical judgements have not been calibrated to assess dynamic risk changes that occur over short time intervals. The ability and reliability of health care professions, no matter what their training or experience, to assess these small changes is unknown. Contiguous changes in risk for ICU patients are generally small (17) and may be dependent on a small changes in a large number of variables. The ability to successfully integrate this large amount of changing information on a frequent basis lies beyond the capabilities of most care givers, especially those who are less experienced (38). Furthermore, the model's high precision of 0.594 resulting in a number needed to evaluate of 1.7 patients coupled with an accuracy of 94.7% suggests that any potential burden of increased patient assessments to an ICU care team is negligible with a high benefit if a model-detected clinical deterioration can lead to earlier clinical intervention. The potential benefit is confirmed by the net benefit analysis (Supplementary Appendix S6: Net Benefit Analysis of the Criticality Index-Mortality Models for both Treated and Untreated Patients). Conversely, with a high negative predictive value of 0.958, the model can potentially be utilized to direct clinical reassessments away from low risk patients, and towards those that need them most; this potential model utilization is particularly important during periods of high census. Therefore, the addition of frequently updated risk assessments for children in ICUs could result in the detection of clinical deterioration or improvement that might have been unappreciated, providing an opportunity for earlier interventions and the potential for improved outcomes.

Construct validity in this study and others was assessed using clinical trajectories, overall and within clinical entities, which were consistent with clinical expectations (17, 28). The validity for the Criticality Index, however, has been further explored. First, the variables based on therapy, physiology and intensity used in the Criticality Index are fundamental to severity of illness. In the 1960′s and 1970′s, The Clinical Classification System was proposed as a measure of severity based on intensity of care and the Therapeutic Intervention Scoring System, a measure of severity based on therapies, was an early quantitative assessment of severity of illness (43). In the 1980′s and 1990′s, physiology-based systems were developed and are the early iterations of the APACHE and PRISM systems (44, 45). Second, the Criticality Index is highly correlated with patients' care areas that are representative of severity of illness (22). Third, clinical instability measured with the Criticality Index demonstrates increased volatility in deaths compared to survivors, consistent with the observations of Yoon et al. (14). And fourth, changes in the Criticality Index over time periods as short as 6 h strongly correlate with clinical deterioration and clinical improvement (17).

There are limitations to this study. First, while there is value in an institution specific algorithm, there is a limitation because single site datasets will usually have a relatively small sample size with the potential for overfitting the model. A larger dataset could have improved our model performance, especially during the time periods of prolonged ICU stays. Second, we did not exhaust all issues and possible modeling approaches available for machine learning. For example, data missingness is a routine issue for many machines learning approaches and, although we did not find any systematic bias, it has the potential to introduce bias and could have influenced the LIME analysis. Third, we were unable to explore the details of the risk trajectories for the specific disease processes because most high-risk conditions are infrequently represented in multi-disciplinary pediatric ICUs. Fourth, we did not use diagnoses for our risk assessments because these were not easily available in the EHR for the individual time intervals. However, the LIME assessment of important variables demonstrated how inclusion of medications and therapies provided proxies for this information. Fifth, this study, as well as other pediatric ICU studies with mortality as the outcome, will usually have relatively few deaths. The AUROC has limited relevance with very unbalanced datasets and some recommend using the AUPRC under these circumstances (24) Sixth, the data set pre-dated the novel coronavirus pandemic and, if used clinically, the algorithm should be re-evaluated and potentially be retrained. This emphasizes the need to frequently evaluate and re-calibrate predictive models, especially if they used for clinical care. Finally, performance metrics for models such as the CI-M that frequently update risk should be assessed with the understanding that the evaluation cohort is changing in time. For example, patients dying early in their ICU stay during the resuscitation phase of illness will generally have substantial physiologic instability while those dying after long ICU stays may have an illness profile of chronic dysfunction.

In conclusion, the Criticality Index-Mortality computing mortality risk every 3 h for pediatric intensive care unit patients has potential to enhance the clinical assessment of severity of illness and clinical care. The methods are applicable to other institutions.

## Data Availability

The datasets presented in this article are not readily available because; The data can be identified to patient cases, and is therefore not appropriate for sharing. Requests to access the datasets should be directed to; apatel4@childrensnational.org.
