# Supplementary material for: The criticality Index-mortality: A dynamic machine learning prediction algorithm for mortality prediction in children cared for in an ICU
Source: Front Pediatr. 2022 Dec 1;10:1023539. doi: 10.3389/fped.2022.1023539 (PMC9752098; doi:10.3389/fped.2022.1023539)
Supplement: Supplementary file 1 [file Datasheet1.docx]

**Appendix 1: Criticality Index Independent Variables**

**Appendix 2: Medication Classification**

**Appendix 4:** **Count of the number of survivors and deaths per 3-hour time period in the training/validation and test datasets**

**Appendix 4: Details of Imputation for Institutional Criticality Index-Mortality Models**

**Appendix 5: Performance metrics for the Criticality Index-Mortality Models at a cut-point of 0.5**

**Appendix 6: Net Benefit Analysis of the Criticality Index-Mortality Models for both Treated and Untreated Patients**

**Appendix 1. Table A: Criticality Index-Mortality Independent Variables**. Modified from: Rivera EAT, Patel AK, Chamberlain JM, Workman TE, Heneghan JA, Redd D, Morizono H, Kim D, Bost JE, Pollack MM. Criticality: A New Concept of Severity of Illness for Hospitalized Children. Pediatr Crit Care Med. 2021 Jan 1;22(1):e33-e43. doi: 10.1097/PCC.0000000000002560. PMID: 32932406; PMCID: PMC7790867.

| **Lab Variables^1,3,4^** | | | | **Vital Signs^1,3,4^** | **Medications^2,3^** | **Other** |
| --- | --- | --- | --- | --- | --- | --- |
| Albumin | Bilirubin Indirect | Hemoglobin | Platelets | BP-systolic | 1113 individual medications | Age  Sex |
| ALT | Bilirubin Total | Hematocrit | Potassium | BP- diastolic | 143 medication categories (6) | Mechanical Ventialiton |
| Arterial Lactate | BUN | INR | Protime | Heart Rate |  |  |
| PO2 (arterial) | Calcium | Glucose | Sodium | Respiratory Rate |  |  |
| AST | Calcium Ionized | PTT | Total Protein | Temperature |  |  |
| Base Excess | Chloride | PCO2 (5) | Venous Lactate | Coma Score |  |  |
| Bicarbonate | Creatinine | pH (5) | WBC |  |  |  |
| Bilirubin Direct | Fibrinogen |  |  |  |  |  |
| 1. Summarized for modeling with the following statistics for each variable: the count, sample mean, sample standard deviation (0 if the count was <2), maximum, and minimum. 2. Summarized for modeling with the following statistics: the 6-hour sum per medication category of the number of medications given each hour; 2) the count of the previous time periods per medication category that the patient received one or more medications; 3) the proportion of the previous time periods per medication category that the patient received one or more medications. 3. Therapeutic intensity is reflected in the number of vital sign and laboratory measurements and medications. 4. If during the first six-hour time period there were missing values, these values were adjusted to the median of the first six-hour time periods adjusted to the following age groups: <1week, 1week-<4weeks, 4weeks-<3months, 3months-<1year, 1year-<2years, 2years-<3years, 3years-<8years, 8years-<12years, 12years-<22years. 5. Arterial, venous, capillary. 6. Classified by Multum. | | | | | |  |

**Appendix 2: Medication Classification**

To assess medications by class, the medication table was linked to an industry standard for medication classification, Multum™ , via the NDC number. ^4,5^ Multum™ provides information about medications therapeutic action in three categories from general mechanism of action to a specific therapeutic category. Table B features the 144 medication categories that utilized Multum™ to categorize 96% of medications. For medications present in multiple Multum™ categories, a prioritization list was developed to classify each medication into the category most relevant to ICU care (Table C).

Some medication categories were combined if they had similar mechanisms of action or therapeutic usage. Inotropic agents, vasopressin, vasopressors and vasopressin antagonists were combined as their mechanism of action targets blood pressure elevation and they often have varying effects at different doses. Immunosuppressive agents, anti-neoplastic and anti-rheumatics were combined as all medications target reducing the body’s innate immune system. All anti-cholinergic categories (anti-Parkinson agents, chronotropic agents and anti-spasmodic agents) were combined due to similar mechanisms of action and the inability to determine which therapeutic purpose they were employed for. Inhaled nitric oxide (iNO) is currently not FDA approved for pulmonary hypertension in pediatric patients; however it is used for this purpose or similar physiologic reasons in the ICU. Therefore iNO was included in the “agents for pulmonary hypertension” category. Muscle relaxants and neuromuscular blockade agents were combined as several drugs are present in both categories. Erythropoiesis stimulating agents and colony stimulating agents were combined as both promote stimulation of cell lines. Cardio-selective and non-cardio selective) beta blockers were combined due to their similar mechanism of action and therapeutic purpose. Anti-depressants and antipsychotics were also combined as both are psychotherapeutic agents employed in the ICU.

The drugs not categorized by Multum™ were categorized via independent assessment by two physicians (AKP, MMP) into the existing categories by their mechanism of action and/or therapeutic purpose (Table D).

**Table B. Original Multum™ Categories Applied to Medication Data**

| **Multum™ Categories** |
| --- |
| Ace inhibitors |
| Ace inhibitors with thiazides |
| Adrenal cortical steroids |
| Agents for hypertensive emergencies |
| Agents for pulmonary hypertension |
| Analgesics |
| Angiotensin ii inhibitors |
| Anorectal preparations |
| Anti infective |
| Antiadrenergic agents centrally acting |
| Antiadrenergic agents peripherally acting |
| Antianginal agents |
| Antiarrhythmic agents |
| Anticholinergic antiparkinson agents |
| Anticholinergic chronotropic agents |
| Anticholinergics antispasmodics |
| Anticoagulant reversal agents |
| Anticoagulants |
| Anticonvulsants |
| Antidepressants |
| Antidiabetic agents |
| Antidiuretic hormones |
| Antidotes |
| Antiemetic antivertigo agents |
| Antigout agents |
| Antihistamines |
| Antihyperlipidemic agents |
| Antihypertensive combinations |
| Antihyperuricemic agents |
| Antineoplastics |
| Antiplatelet Agents |
| Antipsychotics |
| Antirheumatics |
| Antiseptic And Germicides |
| Antitussives |
| Anxiolytics sedatives and hypnotics |
| Beta blockers cardioselective |
| Beta blockers non-cardioselective |
| Biologicals |
| Bisphosphonates |
| Bronchodilators |
| Calcium Channel Blocking Agents |
| Cation Exchange Resins |
| Cerumenolytics |
| Chelating Agents |
| Cholinergic Agonists |
| Cholinergic Muscle Stimulants |
| Cholinesterase Inhibitors |
| CNS Stimulants |
| Coagulation Modifiers |
| Decongestants |
| Diuretics |
| Dopaminergic antiparkinsonism agents |
| Erythropoiesis stimulating agents |
| Expectorants |
| Gastrointestinal agents |
| General anesthetics |
| Glucose elevating agents |
| Hormones hormone modifiers |
| Immunologic agents |
| Immunosuppressive agents |
| Inotropic agents |
| Intravenous nutritional products |
| Ionic iodinated contrast media |
| Iron products |
| Leukotriene modifiers |
| Local injectable anesthetics |
| Lung surfactants |
| Magnetic resonance imaging contrast media |
| Minerals and electrolytes |
| Miscellaneous bone resorption inhibitors |
| Miscellaneous cardiovascular agents |
| Miscellaneous central nervous system agents |
| Miscellaneous coagulation modifiers |
| Miscellaneous diagnostic dyes |
| Miscellaneous genitourinary tract agents |
| Miscellaneous ophthalmic agents |
| Miscellaneous respiratory agents |
| Miscellaneous topical agents |
| Miscellaneous uncategorized agents |
| Miscellaneous vaginal agents |
| Mouth and throat products |
| Muscle relaxants |
| Mydriatics |
| Narcotic analgesics |
| Nasal antihistamines and decongestants |
| Nasal lubricants and irrigations |
| Nasal steroids |
| Neuromuscular blocking agents |
| Nitric oxide |
| Non iodinated contrast media |
| Non ionic iodinated contrast media |
| Nutraceutical products |
| Ophthalmic anesthetics |
| Ophthalmic anti -infectives |
| Ophthalmic anti-inflammatory agents |
| Ophthalmic antihistamines and decongestants |
| Ophthalmic diagnostic agents |
| Ophthalmic glaucoma agents |
| Ophthalmic lubricants and irrigations |
| Ophthalmic steroids |
| Ophthalmic steroids with anti-infectives |
| Ophthalmic surgical agents |
| Oral nutritional supplements |
| Other |
| Otic anti-infectives |
| Otic steroids with anti-infectives |
| Peripheral vasodilators |
| Phosphate binders |
| Plasma expanders |
| Platelet stimulating agents |
| Probiotics |
| Respiratory inhalant products |
| Sclerosing agents |
| Smoking cessation agents |
| Sterile irrigating solutions |
| Topical acne agents |
| Topical anesthetics |
| Topical anti-infectives |
| Topical anti -rosacea agents |
| Topical antibiotics |
| Topical antifungals |
| Topical antihistamines |
| Topical antipsoriatics |
| Topical antivirals |
| Topical astringents |
| Topical debriding agents |
| Topical emollients |
| Topical non-steroidal anti-inflammatories |
| Topical rubefacient |
| Topical steroids |
| Topical steroids with anti-infectives |
| Ultrasound contrast media |
| Upper respiratory combinations |
| Urea cycle disorder agents |
| Urinary antispasmodics |
| Urinary pH modifiers |
| Uterotonic agents |
| Vaginal anti-infectives |
| Vasodilators |
| Vasopressin antagonists |
| Vasopressors |
| Vitamin and mineral combinations |
| Vitamins |

**Table C: Medication Prioritization**

| Neuromuscular Blockade Agents |
| --- |
| Vasopressor |
| Inotropic agents |
| Anxiolytics, Sedatives and Hypnotics |
| Analgesics |
| Antiarrhythmic agents |
| General Anesthetics |
| Anticonvulsant |
| Agents for hypertensive emergencies |
| Beta blockers, cardio-selective |
| Beta blockers, non-cardio-selective |
| Beta blockers with thiazides |
| Calcium Channel Blocking agents |
| Ace-inhibitor with thiazides |
| Ace-inhibitor with calcium channel blocking agents |
| Angiotensin converting enzyme (ACE) inhibitor |
| Angiotensin II inhibitor |
| Angiotensin II inhibitor with thiazide, |
| Angiotensin receptor blockers and neprilysin inhibitors |
| Angiotensin II inhibitors with calcium channel blockers |
| Agents for pulmonary hypertension |
| Anticholinergic chronotropic agents |
| Lung surfactants |
| Anticoagulant |
| Minerals and electrolytes |
| bronchodilators |
| Analgesics |
| Antiplatelet agents |
| Diuretics |
| Antidiabetic agents |
| Anti-infectives |
| Adrenal cortical steroids |
| Hormones/hormone modifiers |
| Diuretics |
| Anti-arrhythmic agents |
| antihypertensive combinations |
| Anti-neoplastic |
| Antidepressant |
| gastrointestinal agents |
| anticholinergics/antispasmodics |
| Antihistamine |
| Immunosuppressive agents |
| Immunologic agents |
| Colony stimulating factors |
| Antihyperuricemic agents |
| Antirheumatics |
| Respiratory inhalant products |
| Antihistamine |
| Hormone/hormone modifiers |
| Antiemetic/antivertigo agents |
| Urinary antispasmodic |
| Intravenous nutritional products |
| Vasodilators |
| Plasma expanders |
| Anticoagulants |
| Antiadrenergic agents, centrally acting |
| Cholinergic muscle stimulants |
| CNS stimulants |
| Antidotes |
| Erythropoiesis-stimulating agents |
| Miscellaneous cardiovascular agents |
| Antidiuretic hormones |
| Glucose elevating agents |
| Muscle relaxants |
| Antiarrhythmic agents |
| Vasopressin antagonists |
| Antihyperlipidemic agents |
| Biologicals |
| Antianginal agents |
| Antineoplastics |
| Cholinesterase inhibitors |
| Nitric oxide |
| Vasopressin |

**Table D: Manually Categorized Medications**

| **Drug Name** | **First Tier Category Name** | **Second Tier Category Name** | **Third Tier Category Name** |
| --- | --- | --- | --- |
| 5-aminosalicylic acid | 5-aminosalicylates | gastrointestinal agents |  |
| Amrinone | inotropic agents | cardiovascular agents |  |
| Botulism antitoxin bivalent (Equine) A and B | antitoxins and antivenins | biologicals |  |
| Bretylium | group III antiarrhythmics | antiarrhythmic agents | cardiovascular agents |
| Carbenicillin | antipseudomonal penicillins | penicillins | anti-infectives |
| Carbetocin | uterotonic agents | genitourinary tract agents |  |
| Cefamandole | second generation cephalosporins | cephalosporins | anti-infectives |
| Cefonicid | second generation cephalosporins | cephalosporins | anti-infectives |
| Ceftizoxime | third generation cephalosporins | cephalosporins | anti-infectives |
| Cephalothin | first generation cephalosporins | cephalosporins | anti-infectives |
| Cilazapril | angiotensin converting enzyme (ACE) inhibitors | cardiovascular agents |  |
| Cilazapril-hydrochlorothiazide | ACE inhibitors with thiazides | antihypertensive combinations | cardiovascular agents |
| Cloxacillin | penicillinase resistant penicillins | penicillins | anti-infectives |
| Danaparoid | heparins | anticoagulants | coagulation modifiers |
| Digitoxin | inotropic agents | cardiovascular agents | NULL |
| Dirithromycin | macrolides | macrolide derivatives | anti-infectives |
| Doxacurium | neuromuscular blocking agents | muscle relaxants | central nervous system agents |
| Elvitegravir | integrase strand transfer inhibitor | antiviral agents | anti-infectives |
| Enalapril-felodipine | ACE inhibitors with calcium channel blocking agents | antihypertensive combinations | cardiovascular agents |
| Enflurane | general anesthetics | central nervous system agents | NULL |
| Fenoterol | adrenergic bronchodilators | bronchodilators | respiratory agents |
| Fenoterol-ipratropium | bronchodilator combinations | bronchodilators | respiratory agents |
| Fomivirsen | miscellaneous antivirals | antiviral agents | anti-infectives |
| Fospropofol | general anesthetics | central nervous system agents | NULL |
| Halofantrine | miscellaneous antimalarials | antimalarial agents | anti-infectives |
| Halothane | general anesthetics | central nervous system agents | NULL |
| Insulin | insulin | antidiabetic agents | metabolic agents |
| Insulin isophane | insulin | antidiabetic agents | metabolic agents |
| Insulin zinc | insulin | antidiabetic agents | metabolic agents |
| Insulin zinc extended | insulin | antidiabetic agents | metabolic agents |
| Interferon alfa-2a | antineoplastic interferons | antineoplastics | NULL |
| Interferon alfa-2b-ribavirin | antiviral combinations | antiviral agents | anti-infectives |
| Interferon alfacon-1 | interferons | immunostimulants | immunologic agents |
| Interferon alfa-n1 | interferons | immunostimulants | immunologic agents |
| Iodoquinol | amebicides | anti-infectives | NULL |
| Ipratropium-salbutamol | bronchodilator combinations | bronchodilators | respiratory agents |
| Isoetharine | adrenergic bronchodilators | bronchodilators | respiratory agents |
| Kanamycin | aminoglycosides | anti-infectives | NULL |
| Ketotifen | ophthalmic antihistamines and decongestants | ophthalmic preparations | topical agents |
| Lipid surfactant | lung surfactants | respiratory agents |  |
| Lomefloxacin | quinolones | anti-infectives | NULL |
| Loracarbef | second generation cephalosporins | cephalosporins | anti-infectives |
| Lucinactant | lung surfactants | respiratory agents | NULL |
| Lymphocyte immune globulin, anti-thymocyte | selective immunosuppressants | immunosuppressive agents | immunologic agents |
| Magaldrate | antacids | gastrointestinal agents | NULL |
| Mephobarbital | barbiturates | anxiolytics, sedatives, and hypnotics | central nervous system agents |
| Mephobarbital | barbiturate anticonvulsants | anticonvulsants | central nervous system agents |
| Metaraminol | vasopressors | cardiovascular agents | NULL |
| Methoxyflurane | general anesthetics | central nervous system agents | NULL |
| Moricizine | group I antiarrhythmics | antiarrhythmic agents | cardiovascular agents |
| Muromonab-CD3 | selective immunosuppressants | immunosuppressive agents | immunologic agents |
| Nadroparin | heparins | anticoagulants | coagulation modifiers |
| Nedocromil | mast cell stabilizers | respiratory inhalant products | respiratory agents |
| Netilmicin | aminoglycosides | anti-infectives | NULL |
| Nicoumalone | coumarins and indanediones | anticoagulants | coagulation modifiers |
| Nitrazepam | benzodiazepine anticonvulsants | anticonvulsants | central nervous system agents |
| Nitroprusside | agents for hypertensive emergencies | cardiovascular agents |  |
| Norfloxacin | quinolones | anti-infectives | NULL |
| Nylidrin | peripheral vasodilators | cardiovascular agents |  |
| Oprelvekin | interleukins | immunostimulants | immunologic agents |
| Oprelvekin | platelet-stimulating agents | coagulation modifiers | NULL |
| Orciprenaline | adrenergic bronchodilators | bronchodilators | respiratory agents |
| Oxprenolol | beta blockers, non-cardioselective | beta-adrenergic blocking agents | cardiovascular agents |
| Paraldehyde | miscellaneous anxiolytics, sedatives and hypnotics | anxiolytics, sedatives, and hypnotics | central nervous system agents |
| Peginesatide | erythropoiesis-stimulating agents | biologicals | NULL |
| Peginterferon alfa-2b-ribavirin | antiviral combinations | antiviral agents | anti-infectives |
| Penbutolol | beta blockers, non-cardioselective | beta-adrenergic blocking agents | cardiovascular agents |
| Penicillin | natural penicillins | penicillins | anti-infectives |
| Pentastarch | miscellaneous uncategorized agents | miscellaneous agents | NULL |
| Pipecuronium | neuromuscular blocking agents | muscle relaxants | central nervous system agents |
| Piperacillin | antipseudomonal penicillins | penicillins | anti-infectives |
| Pirbuterol | adrenergic bronchodilators | bronchodilators | respiratory agents |
| Pivampicillin | aminopenicillins | penicillins | anti-infectives |
| Pivmecillinam | natural penicillins | penicillins | anti-infectives |
| Pizotifen | migraine treatment |  |  |
| Plicamycin | antineoplastic antibiotics | antineoplastics | NULL |
| Poractant | lung surfactants | respiratory agents |  |
| Proguanil | miscellaneous antimalarials | antimalarial agents | anti-infectives |
| Ralitrexed | folate antimetabolite for chemotherapy |  |  |
| Rapacuronium | neuromuscular blocking agents | muscle relaxants | central nervous system agents |
| Respiratory syncytial virus immune globulin | immune globulins | immunologic agents | NULL |
| Reteplase | thrombolytics | coagulation modifiers | NULL |
| Salbutamol | adrenergic bronchodilators | bronchodilators | respiratory agents |
| Simvastatin-sitaGLIPtin | antihyperlipidemic combinations | antihyperlipidemic agents | metabolic agents |
| Sparfloxacin | quinolones | anti-infectives | NULL |
| Spectinomycin | miscellaneous antibiotics | anti-infectives | NULL |
| Spiramycin | macrolides | macrolide derivatives | anti-infectives |
| Stanozolol | androgens and anabolic steroids | sex hormones | hormones/hormone modifiers |
| Streptokinase | thrombolytics | coagulation modifiers | NULL |
| Sulfadiazine-trimethoprim | sulfonamides | anti-infectives |  |
| Sulfamethizole | sulfonamides | anti-infectives | NULL |
| Sulfamethoxazole | sulfonamides | anti-infectives | NULL |
| Sulfapyridine | sulfonamides | anti-infectives |  |
| Sulfisoxazole | sulfonamides | anti-infectives | NULL |
| Thioproperazine | phenothiazine antipsychotics | antipsychotics | psychotherapeutic agents |
| Thyrotropin alpha | thyroid hormones | hormones/hormone modifiers |  |
| Ticarcillin | antipseudomonal penicillins | penicillins | anti-infectives |
| Ticarcillin-clavulanate | penicillins/beta-lactamase inhibitors | penicillins | anti-infectives |
| Trimetrexate | miscellaneous antibiotics | anti-infectives | NULL |

**Appendix 3: Count of the number of survivors and deaths per 3-hour time period in the training/validation and test datasets**

**Table E: Count and percent of the number of survivors and deaths per 3-hour time period in the training and validation dataset**

| Hours in the ICU | Days in the ICU | Count and Percentage of all Survivors^1^ | Count and percentage of all Deaths^2^ |
| --- | --- | --- | --- |
| 3 | 0.125 | 7064 (100.00) | 281 (100.00) |
| 6 | 0.25 | 7042 (99.69) | 280 (99.64) |
| 9 | 0.375 | 6726 (95.22) | 277 (98.58) |
| 12 | 0.5 | 6341 (89.77) | 273 (97.15) |
| 15 | 0.625 | 6080 (86.07) | 270 (96.09) |
| 18 | 0.75 | 5891 (83.39) | 267 (95.02) |
| 21 | 0.875 | 5643 (79.88) | 264 (93.95) |
| 24 | 1 | 5367 (75.98) | 259 (92.17) |
| 27 | 1.125 | 5024 (71.12) | 258 (91.81) |
| 30 | 1.25 | 4642 (65.71) | 255 (90.75) |
| 33 | 1.375 | 4332 (61.33) | 248 (88.26) |
| 36 | 1.5 | 4050 (57.33) | 246 (87.54) |
| 39 | 1.625 | 3790 (53.65) | 241 (85.77) |
| 42 | 1.75 | 3555 (50.33) | 240 (85.41) |
| 45 | 1.875 | 3372 (47.73) | 233 (82.92) |
| 48 | 2 | 3196 (45.24) | 229 (81.49) |
| 51 | 2.125 | 3032 (42.92) | 222 (79.00) |
| 54 | 2.25 | 2873 (40.67) | 216 (76.87) |
| 57 | 2.375 | 2719 (38.49) | 212 (75.44) |
| 60 | 2.5 | 2606 (36.89) | 205 (72.95) |
| 63 | 2.625 | 2490 (35.25) | 202 (71.89) |
| 66 | 2.75 | 2386 (33.78) | 202 (71.89) |
| 69 | 2.875 | 2291 (32.43) | 200 (71.17) |
| 72 | 3 | 2206 (31.23) | 196 (69.75) |
| 75 | 3.125 | 2123 (30.05) | 193 (68.68) |
| 78 | 3.25 | 2047 (28.98) | 188 (66.90) |
| 81 | 3.375 | 1972 (27.92) | 186 (66.19) |
| 84 | 3.5 | 1912 (27.07) | 182 (64.77) |
| 87 | 3.625 | 1840 (26.05) | 179 (63.70) |
| 90 | 3.75 | 1782 (25.23) | 176 (62.63) |
| 93 | 3.875 | 1727 (24.45) | 174 (61.92) |
| 96 | 4 | 1661 (23.51) | 167 (59.43) |
| 99 | 4.125 | 1615 (22.86) | 165 (58.72) |
| 102 | 4.25 | 1565 (22.15) | 163 (58.01) |
| 105 | 4.375 | 1533 (21.70) | 160 (56.94) |
| 108 | 4.5 | 1497 (21.19) | 159 (56.58) |
| 111 | 4.625 | 1466 (20.75) | 156 (55.52) |
| 114 | 4.75 | 1437 (20.34) | 152 (54.09) |
| 117 | 4.875 | 1407 (19.92) | 151 (53.74) |
| 120 | 5 | 1384 (19.59) | 147 (52.31) |
| 123 | 5.125 | 1344 (19.03) | 144 (51.25) |
| 126 | 5.25 | 1311 (18.56) | 142 (50.53) |
| 129 | 5.375 | 1292 (18.29) | 141 (50.18) |
| 132 | 5.5 | 1268 (17.95) | 141 (50.18) |
| 135 | 5.625 | 1249 (17.68) | 140 (49.82) |
| 138 | 5.75 | 1229 (17.40) | 140 (49.82) |
| 141 | 5.875 | 1213 (17.17) | 139 (49.47) |
| 144 | 6 | 1199 (16.97) | 139 (49.47) |
| 147 | 6.125 | 1183 (16.75) | 137 (48.75) |
| 150 | 6.25 | 1160 (16.42) | 133 (47.33) |
| 153 | 6.375 | 1154 (16.34) | 131 (46.62) |
| 156 | 6.5 | 1135 (16.07) | 130 (46.26) |
| 159 | 6.625 | 1121 (15.87) | 128 (45.55) |
| 162 | 6.75 | 1108 (15.69) | 124 (44.13) |
| 165 | 6.875 | 1094 (15.49) | 122 (43.42) |
| 168 | 7 | 1074 (15.20) | 121 (43.06) |
| 171 | 7.125 | 1056 (14.95) | 117 (41.64) |
| 174 | 7.25 | 1041 (14.74) | 117 (41.64) |
| 177 | 7.375 | 1027 (14.54) | 116 (41.28) |
| 180 | 7.5 | 1013 (14.34) | 116 (41.28) |
| 183 | 7.625 | 1002 (14.18) | 115 (40.93) |
| 186 | 7.75 | 994 (14.07) | 113 (40.21) |
| 189 | 7.875 | 979 (13.86) | 113 (40.21) |
| 192 | 8 | 970 (13.73) | 112 (39.86) |
| 195 | 8.125 | 956 (13.53) | 109 (38.79) |
| 198 | 8.25 | 944 (13.36) | 108 (38.43) |
| 201 | 8.375 | 938 (13.28) | 106 (37.72) |
| 204 | 8.5 | 930 (13.17) | 106 (37.72) |
| 207 | 8.625 | 925 (13.09) | 106 (37.72) |
| 210 | 8.75 | 912 (12.91) | 106 (37.72) |
| 213 | 8.875 | 899 (12.73) | 105 (37.37) |
| 216 | 9 | 893 (12.64) | 99 (35.23) |
| 219 | 9.125 | 888 (12.57) | 98 (34.88) |
| 222 | 9.25 | 880 (12.46) | 97 (34.52) |
| 225 | 9.375 | 875 (12.39) | 95 (33.81) |
| 228 | 9.5 | 865 (12.25) | 93 (33.10) |
| 231 | 9.625 | 860 (12.17) | 92 (32.74) |
| 234 | 9.75 | 847 (11.99) | 91 (32.38) |
| 237 | 9.875 | 841 (11.91) | 90 (32.03) |
| 240 | 10 | 827 (11.71) | 89 (31.67) |
| 243 | 10.125 | 818 (11.58) | 88 (31.32) |
| 246 | 10.25 | 805 (11.40) | 88 (31.32) |
| 249 | 10.375 | 798 (11.30) | 88 (31.32) |
| 252 | 10.5 | 792 (11.21) | 88 (31.32) |
| 255 | 10.625 | 786 (11.13) | 88 (31.32) |
| 258 | 10.75 | 776 (10.99) | 87 (30.96) |
| 261 | 10.875 | 771 (10.91) | 86 (30.60) |
| 264 | 11 | 768 (10.87) | 86 (30.60) |
| 267 | 11.125 | 756 (10.70) | 86 (30.60) |
| 270 | 11.25 | 751 (10.63) | 86 (30.60) |
| 273 | 11.375 | 740 (10.48) | 84 (29.89) |

1. Survivors are patients who did not die during their hospital admission. The number of survivors is reduced in subsequent time periods due to discharge from the ICU.
2. Deaths are patients who ultimately died during their hospital stay. The number of deaths is reduced in subsequent time periods when they die.

**Table F: Count and percent of the number of survivors and deaths per 3-hour time period in the test dataset**

| Hours in the ICU | Days in the ICU | Count and Percentage of all Survivors^1^ | Count and percentage of all Deaths^2^ |
| --- | --- | --- | --- |
| 3 | 0.125 | 1020 (100.00) | 33 (100.00) |
| 6 | 0.25 | 1015 (99.51) | 33 (100.00) |
| 9 | 0.375 | 972 (95.29) | 33 (100.00) |
| 12 | 0.5 | 913 (89.51) | 33 (100.00) |
| 15 | 0.625 | 875 (85.78) | 32 (96.97) |
| 18 | 0.75 | 843 (82.65) | 32 (96.97) |
| 21 | 0.875 | 810 (79.41) | 32 (96.97) |
| 24 | 1 | 778 (76.27) | 32 (96.97) |
| 27 | 1.125 | 724 (70.98) | 32 (96.97) |
| 30 | 1.25 | 675 (66.18) | 31 (93.94) |
| 33 | 1.375 | 637 (62.45) | 31 (93.94) |
| 36 | 1.5 | 585 (57.35) | 30 (90.91) |
| 39 | 1.625 | 556 (54.51) | 30 (90.91) |
| 42 | 1.75 | 532 (52.16) | 29 (87.88) |
| 45 | 1.875 | 498 (48.82) | 29 (87.88) |
| 48 | 2 | 474 (46.47) | 28 (84.85) |
| 51 | 2.125 | 444 (43.53) | 26 (78.79) |
| 54 | 2.25 | 414 (40.59) | 26 (78.79) |
| 57 | 2.375 | 395 (38.73) | 26 (78.79) |
| 60 | 2.5 | 372 (36.47) | 23 (69.70) |
| 63 | 2.625 | 358 (35.10) | 23 (69.70) |
| 66 | 2.75 | 343 (33.63) | 23 (69.70) |
| 69 | 2.875 | 328 (32.16) | 23 (69.70) |
| 72 | 3 | 312 (30.59) | 23 (69.70) |
| 75 | 3.125 | 301 (29.51) | 23 (69.70) |
| 78 | 3.25 | 288 (28.24) | 23 (69.70) |
| 81 | 3.375 | 283 (27.75) | 22 (66.67) |
| 84 | 3.5 | 270 (26.47) | 22 (66.67) |
| 87 | 3.625 | 263 (25.78) | 22 (66.67) |
| 90 | 3.75 | 252 (24.71) | 22 (66.67) |
| 93 | 3.875 | 247 (24.22) | 22 (66.67) |
| 96 | 4 | 237 (23.24) | 22 (66.67) |
| 99 | 4.125 | 228 (22.35) | 22 (66.67) |
| 102 | 4.25 | 220 (21.57) | 22 (66.67) |
| 105 | 4.375 | 215 (21.08) | 22 (66.67) |
| 108 | 4.5 | 208 (20.39) | 22 (66.67) |
| 111 | 4.625 | 201 (19.71) | 22 (66.67) |
| 114 | 4.75 | 196 (19.22) | 21 (63.64) |
| 117 | 4.875 | 193 (18.92) | 21 (63.64) |
| 120 | 5 | 189 (18.53) | 21 (63.64) |
| 123 | 5.125 | 183 (17.94) | 20 (60.61) |
| 126 | 5.25 | 179 (17.55) | 20 (60.61) |
| 129 | 5.375 | 177 (17.35) | 20 (60.61) |
| 132 | 5.5 | 174 (17.06) | 20 (60.61) |
| 135 | 5.625 | 172 (16.86) | 20 (60.61) |
| 138 | 5.75 | 171 (16.76) | 20 (60.61) |
| 141 | 5.875 | 168 (16.47) | 20 (60.61) |
| 144 | 6 | 168 (16.47) | 19 (57.58) |
| 147 | 6.125 | 164 (16.08) | 19 (57.58) |
| 150 | 6.25 | 164 (16.08) | 18 (54.55) |
| 153 | 6.375 | 160 (15.69) | 18 (54.55) |
| 156 | 6.5 | 155 (15.20) | 17 (51.52) |
| 159 | 6.625 | 154 (15.10) | 17 (51.52) |
| 162 | 6.75 | 150 (14.71) | 17 (51.52) |
| 165 | 6.875 | 149 (14.61) | 16 (48.48) |
| 168 | 7 | 148 (14.51) | 16 (48.48) |
| 171 | 7.125 | 148 (14.51) | 16 (48.48) |
| 174 | 7.25 | 144 (14.12) | 15 (45.45) |
| 177 | 7.375 | 142 (13.92) | 15 (45.45) |
| 180 | 7.5 | 139 (13.63) | 15 (45.45) |
| 183 | 7.625 | 139 (13.63) | 15 (45.45) |
| 186 | 7.75 | 133 (13.04) | 15 (45.45) |
| 189 | 7.875 | 132 (12.94) | 15 (45.45) |
| 192 | 8 | 130 (12.75) | 15 (45.45) |
| 195 | 8.125 | 128 (12.55) | 15 (45.45) |
| 198 | 8.25 | 124 (12.16) | 15 (45.45) |
| 201 | 8.375 | 124 (12.16) | 15 (45.45) |
| 204 | 8.5 | 124 (12.16) | 15 (45.45) |
| 207 | 8.625 | 124 (12.16) | 15 (45.45) |
| 210 | 8.75 | 124 (12.16) | 14 (42.42) |
| 213 | 8.875 | 122 (11.96) | 14 (42.42) |
| 216 | 9 | 121 (11.86) | 14 (42.42) |
| 219 | 9.125 | 120 (11.76) | 14 (42.42) |
| 222 | 9.25 | 117 (11.47) | 13 (39.39) |
| 225 | 9.375 | 116 (11.37) | 13 (39.39) |
| 228 | 9.5 | 115 (11.27) | 13 (39.39) |
| 231 | 9.625 | 111 (10.88) | 13 (39.39) |
| 234 | 9.75 | 111 (10.88) | 13 (39.39) |
| 237 | 9.875 | 111 (10.88) | 13 (39.39) |
| 240 | 10 | 107 (10.49) | 13 (39.39) |
| 243 | 10.125 | 102 (10.00) | 13 (39.39) |
| 246 | 10.25 | 102 (10.00) | 13 (39.39) |
| 249 | 10.375 | 101 (9.90) | 13 (39.39) |
| 252 | 10.5 | 100 (9.80) | 13 (39.39) |
| 255 | 10.625 | 100 (9.80) | 13 (39.39) |
| 258 | 10.75 | 100 (9.80) | 13 (39.39) |
| 261 | 10.875 | 100 (9.80) | 13 (39.39) |
| 264 | 11 | 99 (9.71) | 12 (36.36) |
| 267 | 11.125 | 98 (9.61) | 12 (36.36) |
| 270 | 11.25 | 98 (9.61) | 11 (33.33) |
| 273 | 11.375 | 97 (9.51) | 10 (30.30) |

1. Survivors are patients who did not die during their hospital admission. The number of survivors is reduced in subsequent time periods due to discharge from the ICU.
2. Deaths are patients who ultimately died during their hospital stay. The number of deaths is reduced in subsequent time periods when they die.

**Appendix 3. Details of Imputation for Institutional Criticality Index-Mortality Models**

The initial time period required values for all laboratory and vital sign data. We imputed the values from the medians by age groups of those patients who had these measurements in the first time period. Note, that the imputed data also included the measurement count for the laboratory test or vital sign measurement which was set to 0 to indicate an imputed value. The age groups were a composite of the age groups used for display of normal data by various sources.

**Table G. Percentage of initial time periods with imputed values.**

| **Laboratory Variables** | **Percentage** |
| --- | --- |
| Albumin | 76.2 |
| ALT | 77.3 |
| AST | 77.3 |
| PO2 | 98.6 |
| Base Excess | 88.5 |
| Bicarbonate | 59.7 |
| Bilirubin Indirect | 99.8 |
| Bilirubin Total | 76.4 |
| BUN | 59.5 |
| Calcium | 60.4 |
| Calcium Ionized | 89.2 |
| Chloride | 59.1 |
| Creatinine | 59.6 |
| Fibrinogen | 99.3 |
| Glucose | 56.4 |
| Hematocrit | 51.3 |
| Hemoglobin | 50.9 |
| INR | 94.4 |
| Lactate Arterial | 98.8 |
| Lactate Venous | 89.9 |
| PCO2 | 88.5 |
| pH | 88.5 |
| Platelets | 54.6 |
| Potassium | 98.6 |
| Protime | 57.8 |
| PTT | 94.5 |
| Sodium | 57.0 |
| Total Protein | 76.8 |
| White blood count | 53.9 |
|  |  |
| **Vital Signs** |  |
| Blood Pressure Systolic | 12.5 |
| Blood Pressure Diastolic | 12.6 |
| Coma Score | 82.6 |
| Hearth Rate | 0.3 |
| Respiratory Rate | 0.4 |
| Temperature | 6.2 |

ALT = alanine transaminase; AST = aspartate aminotransferase; BUN = blood urea nitrogen; INR = international normalized ratio.

**Table H. Laboratory Data Imputed in the First Time Period.** Values presented are medians.

| **Age Groups** | **Albumin** | **ALT** | **Arterial pO2** | **AST** | **Base Excess** |
| --- | --- | --- | --- | --- | --- |
| **1hr - <1wk** | 2.8 | 15 | 68 | 54 | -4 |
| **1wk - <4wks** | 3.1 | 22 | 61.5 | 39 | -0.2 |
| **4wks - <3mo** | 3.3 | 25 | 61 | 39 | 0.5 |
| **3mo - <1yr** | 3.6 | 26 | 77.5 | 43 | -0.9 |
| **1yr - <2yrs** | 3.8 | 23 | 91 | 43 | -3.9 |
| **2yrs - <3yrs** | 3.8 | 22 | 119 | 41 | -2 |
| **3yrs - <8yrs** | 3.8 | 19 | 113 | 37 | -2 |
| **8yrs - <12yrs** | 3.9 | 19 | 129 | 30 | -2.75 |
| **12yrs - <22yrs** | 3.8 | 20 | 139 | 25 | -3 |
|  |  |  |  |  |  |
|  | **Direct bilirubin** | **Indirect bilirubin** | **Total bilirubin** | **BUN** | **Calcium** |
| **1hr - <1wk** | 0.4 | 6 | 7.25 | 9 | 9 |
| **1wk - <4wks** | 0.4 | 4.6 | 2.25 | 9 | 9.9 |
| **4wks - <3mo** | 0.3 | 0.9 | 0.5 | 9 | 9.8 |
| **3mo - <1yr** | 0.1 | 0.4 | 0.3 | 9 | 9.8 |
| **1yr - <2yrs** | 0.1 | 0.3 | 0.3 | 11 | 9.6 |
| **2yrs - <3yrs** | 0.1 | 0.4 | 0.3 | 11 | 9.5 |
| **3yrs - <8yrs** | 0.1 | 0.4 | 0.4 | 11 | 9.4 |
| **8yrs - <12yrs** | 0.2 | 0.5 | 0.4 | 11 | 9.3 |
| **12yrs - <22yrs** | 0.2 | 0.5 | 0.4 | 11 | 9 |
|  |  |  |  |  |  |
|  | **Ionized Calcium** | **Chloride** | **Creatinine** | **Fibrinogen** | **Glucose** |
| **1hr - <1wk** | 1.2 | 106 | 0.64 | 201.5 | 72 |
| **1wk - <4wks** | 1.3 | 104 | 0.41 | 272.85 | 87.5 |
| **4wks - <3mo** | 1.32 | 105 | 0.33 | 265 | 94.5 |
| **3mo - <1yr** | 1.26 | 104 | 0.32 | 307.5 | 101 |
| **1yr - <2yrs** | 1.2 | 104 | 0.31 | 275 | 97 |
| **2yrs - <3yrs** | 1.24 | 104 | 0.33 | 262 | 96 |
| **3yrs - <8yrs** | 1.2 | 104 | 0.4 | 324.5 | 100 |
| **8yrs - <12yrs** | 1.17 | 103 | 0.5 | 333.5 | 104 |
| **12yrs - <22yrs** | 1.14 | 104 | 0.7 | 341.5 | 105 |
|  |  |  |  |  |  |
|  | **HCO3** | **Hematocrit** | **Hemoglobin** | **INR** | **Arterial Lactate** |
| **1hr - <1wk** | 23 | 47.5 | 16.2 | 1.29 | 1.43 |
| **1wk - <4wks** | 24 | 39.55 | 13.6 | 1.31 | 1.19 |
| **4wks - <3mo** | 24.8 | 31.05 | 10.6 | 1.08 | 1.81 |
| **3mo - <1yr** | 22.45 | 33.8 | 11.4 | 1.26 | 1 |
| **1yr - <2yrs** | 21 | 34.3 | 11.5 | 1.21 | 1.22 |
| **2yrs - <3yrs** | 22 | 34.1 | 11.6 | 1.09 | 1 |
| **3yrs - <8yrs** | 23 | 34.5 | 11.8 | 1.25 | 1.7 |
| **8yrs - <12yrs** | 24 | 36.3 | 12.4 | 1.14 | 1.85 |
| **12yrs - <22yrs** | 24 | 37.3 | 12.6 | 1.2 | 2.2 |
|  |  |  |  |  |  |
|  | **Venous Lactate** | **Partial Thromboplastin Time (PTT)** | **pCO2** | **pH** | **Platelet Count** |
| **1hr - <1wk** | 2.35 | 42.4 | 43.1 | 7.31 | 227 |
| **1wk - <4wks** | 3.15 | 39 | 44.3 | 7.35 | 338 |
| **4wks - <3mo** | 3.77 | 35.15 | 50 | 7.34 | 378 |
| **3mo - <1yr** | 2.2 | 32 | 45 | 7.35 | 339 |
| **1yr - <2yrs** | 1.8 | 29 | 39.6 | 7.35 | 313 |
| **2yrs - <3yrs** | 1.3 | 29 | 39.05 | 7.34 | 290 |
| **3yrs - <8yrs** | 2 | 29 | 39 | 7.35 | 274 |
| **8yrs - <12yrs** | 2.3 | 29 | 37 | 7.32 | 266 |
| **12yrs - <22yrs** | 2.2 | 28.4 | 36 | 7.33 | 239 |
|  |  |  |  |  |  |
|  | **Potassium** | **Pro Time** | **Sodium** | **Total Protein** | **White Blood Cell Count** |
| **1hr - <1wk** | 4.6 | 15.1 | 139 | 5.2 | 13.9 |
| **1wk - <4wks** | 5 | 14.4 | 138 | 5.5 | 11.3 |
| **4wks - <3mo** | 5 | 13.8 | 138 | 5.5 | 10.85 |
| **3mo - <1yr** | 4.6 | 14 | 138 | 6.1 | 11.3 |
| **1yr - <2yrs** | 4.3 | 12.4 | 138 | 6.8 | 12 |
| **2yrs - <3yrs** | 4.2 | 12.35 | 138 | 6.8 | 10.4 |
| **3yrs - <8yrs** | 4 | 12.4 | 138 | 6.9 | 10.2 |
| **8yrs - <12yrs** | 4 | 12.4 | 138 | 7.1 | 9.7 |
| **12yrs - <22yrs** | 3.9 | 13 | 139 | 7 | 10.5 |

**Table I. Vital Sign Values Imputed in the First Time Period.** Values presented are medians.

| **Age Group** | **Systolic BP** | **Diastolic BP** | **Coma Score** | **Heart Rate** | **Respiratory Rate** | **Temperature (Centigrade)** |
| --- | --- | --- | --- | --- | --- | --- |
| **1hr - <1wk** | 63 | 35 | 14 | 150 | 48 | 36.8 |
| **1wk - <4wks** | 76 | 45 | 14 | 159 | 41 | 36.9 |
| **4wks - <3mo** | 87 | 49 | 14 | 158 | 40 | 36.89 |
| **3mo - <1yr** | 99 | 57 | 14 | 148 | 38 | 36.83 |
| **1yr - <2yrs** | 107 | 64 | 15 | 141 | 30 | 37.1 |
| **2yrs - <3yrs** | 108 | 64 | 15 | 133 | 27 | 36.94 |
| **3yrs - <8yrs** | 106 | 64 | 15 | 120 | 24 | 37 |
| **8yrs - <12yrs** | 112 | 67 | 15 | 106 | 21 | 36.9 |
| **12yrs - <22yrs** | 120 | 70 | 15 | 96 | 19 | 36.8 |

Sources for Age References

<https://testdirectory.questdiagnostics.com/test/test-detail/6631/?cc=MASTER>.

<https://www.accp.com/docs/sap/Lab_Values_Table_PedSAP.pdf>.

<https://www.unboundmedicine.com/harrietlane/view/Harriet_Lane_Handbook/309269/all/TABLE_27_1:_Reference_Values>.

<https://pdfs.semanticscholar.org/7106/09b4b2d315e448b4267a49420e1080da25eb.pdf>.

[file:///Q:/PediatricBloodGasesCriticalCarePanelTransportandECMOAgeRelatedReferenceandcv103117%20(2).pdf](file:///Q:\PediatricBloodGasesCriticalCarePanelTransportandECMOAgeRelatedReferenceandcv103117%20(2).pdf).

**Appendix 5: Table J: Performance metrics for the Criticality Index-Mortality Models at a cut-point of 0.5**

| **Sensitivity (1)** | **Precision (2)** | **Accuracy (3)** | **Specificity (4)** | **Negative Predictive Value (6)** | **F1 score (6)** | **MCC (7)** |
| --- | --- | --- | --- | --- | --- | --- |
| 0.304,  (0.288, 0.320) | 0.594, (0.569,0.617) | 0.947, (0.945,0.949) | 0.987, (0.986,0.988) | 0.958, (0.956,0.960) | 0.402, (0.388,0.416) | 0.401, (0.375,0.425) |

1. Sensitivity = true positive rate
2. Precision = Positive predictive value = true positives/[true positive + false positive]. Number needed to evaluate = 1/precision.
3. Accuracy = (true positives + true negatives)/(positives + negatives)
4. Specificity = true negatives
5. Negative predictive value = true negatives/[true negatives + false negatives]
6. The F**_1_** score is a measure of accuracy with a maximum score of 1. It is the harmonic mean of precision and sensitivity.
7. MCC = Mathew’s Correlation Coefficient is used in machine learning to measure the quality of binary classifications between observed and predicted values, with a maximum score of 1.

**Appendix 6: Net Benefit Analysis of the Criticality Index-Mortality Models for both Treated and Untreated Patients**


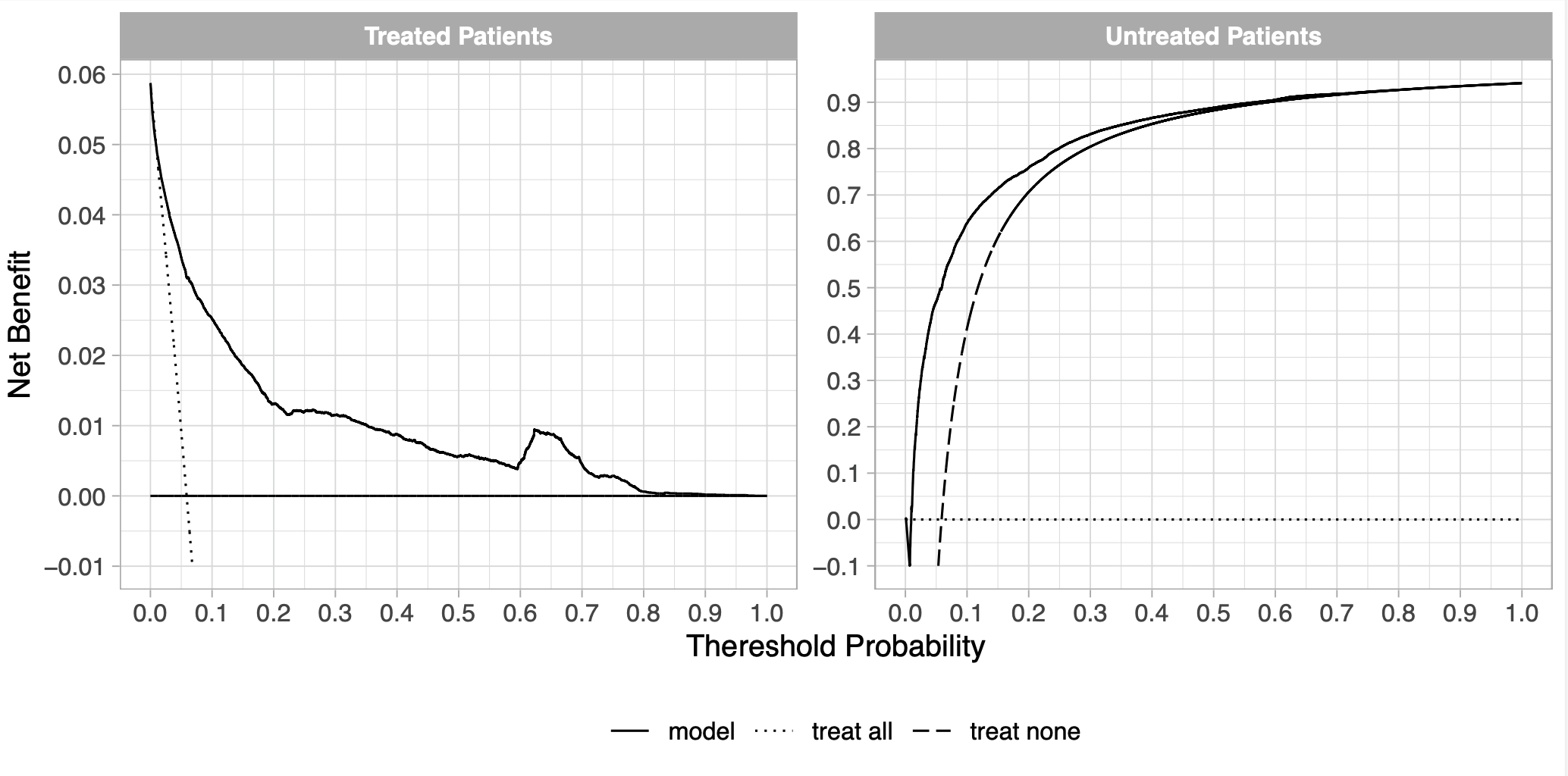


The net benefit analysis^1^ was performed to assess the benefit of applying the Criticality Index-Mortality (CI-M). The benefit on treated patients (left panel) and untreated patients (right panel) is displayed across the full range of probability thresholds. “Treat all” dashed line refers to increased clinical assessments for all patients whereas the “Model” solid line refers to clinical assessments as indicated by the CI-M model (left panel). The “Treat None” dashed line refers to performing no increased clinical assessments to patients as compared to the “Model” solid line clinical assessments as indicated by the CI-M model (right panel).

1. Rousson V, Zumbrunn T. Decision curve analysis revisited: overall net benefit, relationships to ROC curve analysis, and application to case-control studies. BMC Med Inform Decis Mak 2011;11. https://doi.org/10.1186/1472-6947-11-45.
